# Neurofilament light chain and tau concentrations are markedly increased in the serum of patients with sporadic Creutzfeldt-Jakob disease, and tau correlates with rate of disease progression

**DOI:** 10.1136/jnnp-2017-317793

**Published:** 2018-02-27

**Authors:** Andrew Geoffrey Bourne Thompson, Connie Luk, Amanda J Heslegrave, Henrik Zetterberg, Simon H Mead, John Collinge, Graham S Jackson

**Affiliations:** 1 MRC Prion Unit at UCL, UCL Institute of Prion Diseases, London, UK; 2 NHS National Prion Clinic, National Hospital for Neurology and Neurosurgery, University College London Hospitals NHS Foundation Trust, London, UK; 3 Department of Molecular Neuroscience, UCL Institute of Neurology, London, UK; 4 UK Dementia Research Institute at UCL, London, UK; 5 Department of Psychiatry and Neurochemistry, Institute of Neuroscience and Physiology, The Sahlgrenska Academy, University of Gothenburg, Sahlgrenska University Hospital, Mölndal, Sweden; 6 Clinical Neurochemistry Laboratory, Sahlgrenska University Hospital, Mölndal, Sweden

**Keywords:** prion disease, Creutzfeldt-Jakob Disease, tau, neurofilament light, biomarker

## Abstract

**Objectives:**

A blood-based biomarker of neuronal damage in sporadic Creutzfeldt-Jakob disease (sCJD) will be extremely valuable for both clinical practice and research aiming to develop effective therapies.

**Methods:**

We used an ultrasensitive immunoassay to measure two candidate biomarkers, tau and neurofilament light (NfL), in serum from patients with sCJD and healthy controls. We tested longitudinal sample sets from six patients to investigate changes over time, and examined correlations with rate of disease progression and associations with known phenotype modifiers.

**Results:**

Serum concentrations of both tau and NfL were increased in patients with sCJD. NfL distinguished patients from controls with 100% sensitivity and 100% specificity. Tau did so with 91% sensitivity and 83% specificity. Both tau and NfL appeared to increase over time in individual patients, particularly in those with several samples tested late in their disease. Tau, but not NfL, was positively correlated with rate of disease progression, and was particularly increased in patients homozygous for methionine at codon 129 of *PRNP*.

**Conclusions:**

These findings independently replicate other recent studies using similar methods and offer novel insights. They show clear promise for these blood-based biomarkers in prion disease. Future work should aim to fully establish their potential roles for monitoring disease progression and response to therapies.

## Introduction

Sporadic Creutzfeldt-Jakob disease (sCJD), the most common human prion disease, is a progressive and fatal neurodegenerative condition, for which no proven disease-modifying treatment is currently available. Several clinical trials have been performed,^1–4^ other compounds are in preclinical development^5^ and humanised anti-prion protein monoclonal antibodies have been developed for clinical use,^6 7^ but there are a number of major challenges to the design of therapeutic trials in sCJD.

The illness is rapidly progressive and clinically heterogeneous, making the design and implementation of clinical outcome measures challenging. However, progress has been made with designing optimised outcome measures^8^ and using stratification to reduce the impact of heterogeneity.^9^ Patients are often only diagnosed (and offered enrolment into clinical research) at an advanced stage of disease, by which time therapeutic agents may have less potential for benefit. Currently available laboratory biomarkers of sCJD are based on analysis of cerebrospinal fluid (CSF),^10 11^ limiting their potential for serial measurement due to the requirement for lumbar puncture.

A blood-based laboratory biomarker could address several of these challenges. It may allow early identification of potential cases before more involved and invasive investigations have been performed (eg, MRI, lumbar puncture). It may be able to complement clinical outcome measures by demonstrating a biological effect of a therapeutic agent with greater power, allowing better decision-making about advancing from early to larger clinical trials.

Historically, laboratory methods for diagnosing sCJD have relied on intraneuronal proteins that can be detected in the CSF when there is rapid neurodegeneration: the 14-3-3 protein and more recently tau.^10 12^ However, these have now been largely superseded by methods based on in vitro seeded protein misfolding, particularly the real-time quaking-induced conversion assay.^11^ This has been shown to have a sensitivity of between 80% and 91% and specificity of between 98% and 100% for sCJD in different studies,^13–15^ although the levels of seeding activity would be predicted to be largely invariant in the latter stages of disease,^16 17^ so its utility as a progression biomarker may be limited.

Measurement of brain-derived proteins in blood as potential biomarkers of neurodegeneration has been limited by their very low concentrations, often below the limit of detection of standard ELISA and other available assay methods. Recently, however, the use of novel ultrasensitive assay techniques, such as the single molecule array (Simoa) platform (Quanterix, Boston, MA, USA), has allowed measurement of such proteins in blood with limits of detection well below physiological concentrations seen in healthy controls, and this has provided valuable new biomarkers with emerging diagnostic, prognostic and therapy-monitoring roles in a range of different diseases.^18–26^

Tau is an intraneuronal, microtubule-associated protein, which can be released when there is neuronal damage, and also plays a more specific role in the pathogenesis of various neurodegenerative conditions.^27^ Its concentration is known to be markedly increased in the CSF of patients with sCJD, and this correlates with markers of disease severity.^28^ A small study using a standard ELISA to measure serum tau suggested an increase in sCJD compared with controls.^29^ Its concentration in blood, measured using Simoa, has now been shown to have potential as a biomarker in Alzheimer’s disease,^18–20^ as well as in non-degenerative conditions such as hypoxic and traumatic brain injury.^21 22^

The neurofilament light (NfL) chain is a component of the neuronal cytoskeleton. It is released by neuronal, and particularly axonal, damage in a number of diseases, and its concentration can be measured in the CSF, and in blood using Simoa. It has been shown to have a role as a biomarker in diseases including frontotemporal dementia (where concentrations in CSF and serum correlate with clinical and radiological measures of disease ‘intensity’)^23 24^ and multiple sclerosis (where its concentration in CSF and possibly in blood has a role in monitoring subclinical disease activity during disease-modifying treatment).^25 26^

In this study, we selected these two candidate blood-based biomarkers, tau and NfL, and used the Simoa platform to measure their concentrations in serum samples from patients with sCJD and from healthy control volunteers.

All of the patients included in this study were also enrolled in the National Prion Monitoring Cohort, a large prospective natural history study. Using clinical rating scale data collected through this study, we have developed and validated a bespoke rating scale that measures functional decline in patients with sCJD (the MRC Prion Disease Rating Scale (‘MRC Scale’)),^8^ and used this to model linear slopes of functional decline for individual patients.^9^ This allows us to analyse for correlations between biomarkers and rate of clinical disease progression.

Recently, two other groups have reported studies using Simoa to measure tau and NfL in blood in cohorts including patients with sCJD.^30 31^ These included 33 and 65 patients with sCJD, respectively. Both showed that the concentrations of both proteins were elevated in patients with sCJD relative to controls. This provides the opportunity for direct comparison with our results, with the potential for independent replication of findings which is particularly important given the relatively small size of the individual studies. We also extend the analysis by including longitudinal samples from six patients with sCJD, spanning between 49 and 685 days in individual patients, to explore changes over time during the disease process.

## Methods

### Patients, samples and clinical data

Serum samples from 45 patients with sCJD and 24 healthy control volunteers were tested. All cases and controls were enrolled in the National Prion Monitoring Cohort,^8^ and samples were taken at the time of clinical research assessments for that study, using standardised methods for venepuncture, transport, fractionation and storage. Samples were stored at −80°C, and thawed immediately prior to testing. All sCJD cases had either definite or probable sCJD, according to standard diagnostic criteria.^12^ Healthy volunteers were friends, spouses or relatives of patients. Demographic and basic clinical information about the cases and controls is given in table 1. The ‘MRC Scale’ gives a measure of disease severity at the time each sample was taken, running from 20 (no significant impairment of function) to 0 (severely impaired, bedbound, unable to communicate or swallow).^8^ We have used linear mixed modelling of MRC Scale data to model functional decline in patients with sCJD, and we can use this to derive a slope coefficient representing the percentage loss of function per day for an individual patient. The full methods for this are reported elsewhere.^9^ Modelled slopes of decline calculated in this way are available for 23 of the patients with sCJD included in this biomarker study (the others were already at a very advanced stage of functional impairment at their first assessment, making modelling of a meaningful slope impossible).

**Table 1:** Demographic and clinical information for patients with sCJD and healthy controls included in the study

	Patients with sCJD	Healthy controls
N	45	24
Diagnosis		
Definite	40	–
Probable	5	–
*PRNP* codon 129		
MM	21	–
MV	15	–
VV	7	–
Not tested	2	–
Age (at first sample) (n=45)		
Mean	61.3	50.3
SD	8.29	10.34
Range	41.3–81.8	33.7–68.0
Gender		
Male	22	14
Female	23	10
MRC Scale (n=45)		
Median	5	20
Range	0–19	20–20
Days after reported symptom onset (n=45)		
Median	136	–
Range	45–862	–
Days before death (n=44)		
Median	18.5	–
Range	0–1120	–

MRC Scale, MRC Prion Disease Rating Scale; sCJD, sporadic Creutzfeldt-Jakob disease.

A further 16 samples from six of these patients (giving serial sample sets of between two and five samples) were also tested to explore changes in the protein concentrations over time in individual patients. Only the earliest available sample from each patient was included in the main comparison of patients with controls.

### Measurement of tau and NfL concentrations

The Simoa HD-1 analyser platform (Quanterix) was used to measure tau and NfL concentrations using the manufacturer’s tau and NfL reagent kits and according to the manufacturer’s instructions. Samples were introduced into the analyser in 96-well plates, sealed with a preperforated ‘*X-Pierce*’ film (Excel Scientific) to reduce the chance of spillage or aerosol formation.

In the analyser, patient serum samples are diluted fourfold and then incubated with paramagnetic beads coated with anti-tau or anti-NfL antibodies and biotinylated detector antibodies. Beads are then washed and combined with a conjugate of streptavidin-β-galactosidase. This enzyme binds to the biotinylated antibodies, labelling the captured protein molecules of interest. After being washed again, the beads are suspended in a resorufin-β-D-galactopyranoside (RGP) substrate and transferred into an array of microwells, with individual beads being sealed within individual microwells. If the enzyme-labelled protein of interest is bound to a bead it hydrolyses RGP and produces sufficient fluorescent signal to be detected by the analyser, even if only a single molecule is bound. The analyser measures the proportion of ‘positive’ wells containing beads bound to at least one molecule of interest (giving a ‘digital’ output proportional to the amount of the protein of interest in the sample when it is at low concentrations) and also the total fluorescent signal from all wells (giving an ‘analogue’ output proportional to the amount of the protein of interest present in the sample when it is at higher concentrations).

A calibration series containing known concentrations of the protein of interest, spanning both the ‘digital’ and ‘analogue’ ranges, is tested alongside the test samples. A four-parameter logistic curve fit data reduction method is used to generate a calibration curve, which is then used to calculate concentrations of the protein of interest in the test samples.

### Quality control

Samples were tested in duplicate (two aliquots automatically aspirated from a single plate well), and the average of the two readings was used for analysis.

For the tau assay, the mean coefficient of variation (CV) between duplicates was 4.6%. In a subset of 16 samples only a single measurement was produced (technical data from the analyser showed that it had failed to aspirate a second aliquot correctly). As the variation between duplicate readings for other samples was very low, and the technical data identified no problems with the individual measurements from these samples, they were included in the analysis. All samples were tested from a single plate.

For the NfL assay, the mean CV between duplicates was 4.7%. Duplicate measurements were obtained for all samples. Samples were split between two plates and processed together in a single analyser run using the same reagents. sCJD and control samples were randomly distributed across both plates. All serial sample sets from individual patients with sCJD were tested on the same plate to reduce the impact of any minor variation between plates. Two control samples of known NfL concentration (10 and 200 pg/mL) were included on both plates and compared (CVs of 15% and 11%, respectively).

### Statistical analysis

Data were analysed using Microsoft Excel and SPSS. (V22.0) Neither tau nor NfL was normally distributed (Kolmogorov-Smirnov tests, P<0.05). If natural log transformation was applied, log-tau and log-NfL were both plausibly normally distributed within the control group and within the sCJD group (Kolmogorov-Smirnov tests, P>0.05). We therefore analysed raw values using non-parametric statistics and log-transformed values using parametric statistics. Specific tests are given below.

## Results

### Tau

**Figure 1:**
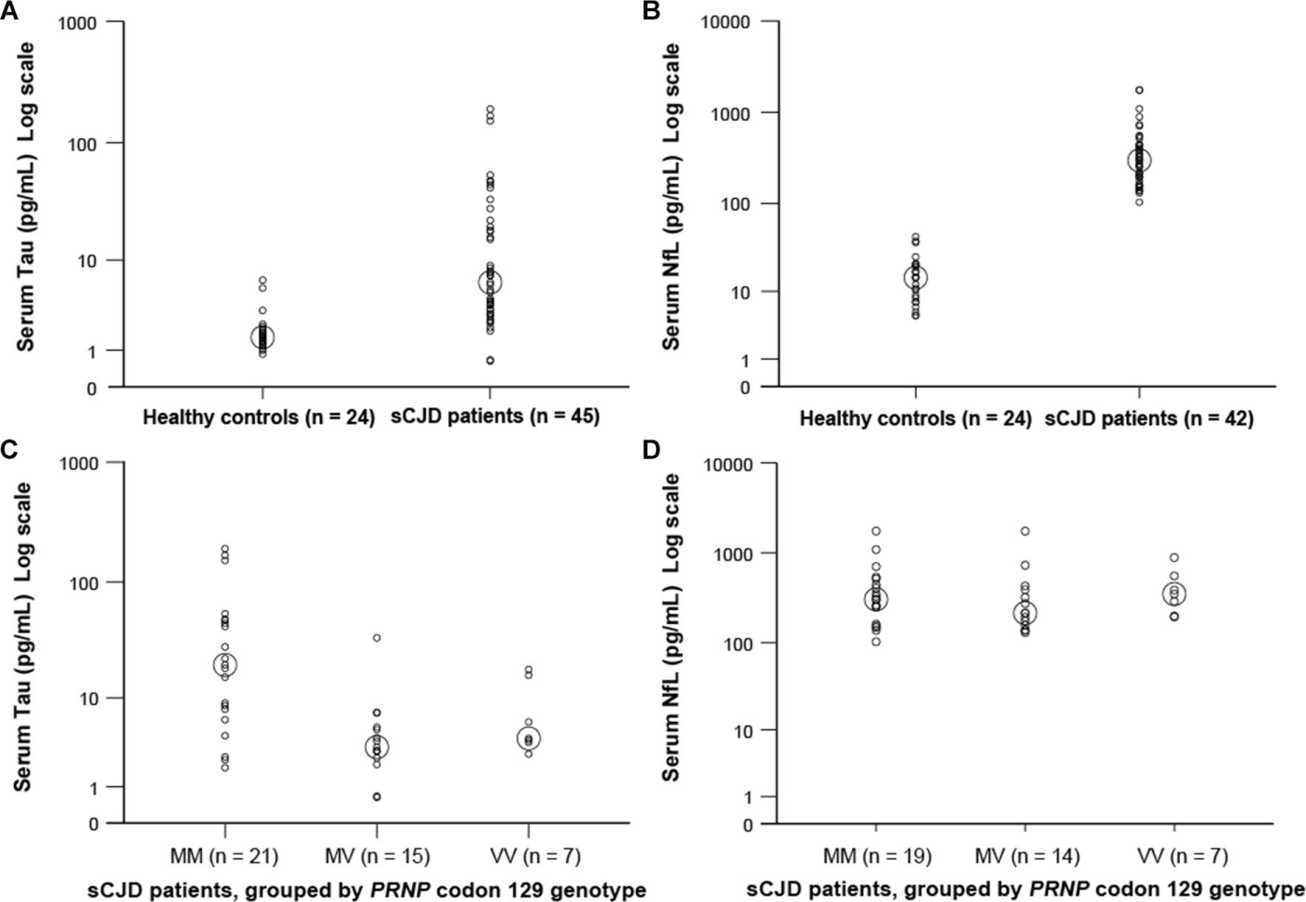
Serum tau and neurofilament light (NfL) concentrations in healthy controls and patients with sporadic Creutzfeldt-Jakob disease (sCJD), and in different sCJD subgroups according to genotype at *PRNP* codon 129. All results are plotted on a log scale. Small circles represent individual sample results (average of two duplicate readings for each sample—see the Methods section). Larger circles indicate median values for each group.

Tau concentrations were higher in patients with sCJD than in healthy controls: median 6.22 pg/mL vs 1.56 pg/mL (Mann-Whitney U test, P<0.001; table 2, figure 1A). Linear regression modelling of the effect of diagnosis (sCJD vs control), age and gender on log-tau showed a strong independent effect of sCJD diagnosis after adjustment for age and gender (β=0.551, P<0.001. r^2^ for model 0.345). Neither age nor gender had independent effects (P=0.95 and P=0.1 respectively).

**Table 2:** Median and IQR for serum tau and NfL concentrations in controls and patients with sCJD. Results for sCJD subgroups according to *PRNP* codon 129 genotype are also shown

		Serum tau (pg/mL)			Serum NfL (pg/mL)		
		n	Median	IQR	n	Median	IQR
Controls		24	**1.56**	1.32–1.97	24	**14.52**	8.04–20.36
sCJD Codon 129	All	45	**6.22**	3.26–19.6	42	**296.6**	193–436
	MM	21	19.6	7.82–47.3	19	305.6	206–480
	MV	15	3.27	2.71–5.12	14	215.1	161–374
	VV	7	4.05	3.78–17.9	7	350.1	245–472

NfL, neurofilament light; sCJD, sporadic Creutzfeldt-Jakob disease.

Median results (shown in bold) and IQR for serum tau and NfL concentrations in controls and patients with sCJD. Results for sCJD subgroups according to *PRNP* codon 129 genotype are also shown.

Tau concentrations were higher in patients with sCJD homozygous for methionine at codon 129 of *PRNP* than in other genotypes: median 19.6 pg/mL in MM, 3.27 pg/mL in MV and 4.05 pg/mL in VV groups (table 2, figure 1C). On linear regression codon 129 genotype had an independent effect on log-tau in sCJD cases after adjustment for age and gender (β=0.434, P=0.004. r^2^ for model 0.22).

### Neurofilament light

NfL concentrations were substantially higher in patients with sCJD than in healthy controls: median 296 pg/mL vs 14.5 pg/mL (Mann-Whitney U test, P<0.001; table 2, figure 1B). Linear regression modelling of the effect of diagnosis (sCJD vs control), age and gender on log-NfL showed a very strong independent effect of sCJD diagnosis after adjustment for age and gender (β=0.829, P<0.001. r^2^ for model 0.873). Age also had an independent effect on log-NfL (β=0.158, P=0.004), as has been seen in other studies.^25 32^ Gender did not appear to have an independent effect (P=0.074).

NfL concentrations did not vary between patients with sCJD with different genotypes at codon 129 (table 2, figure 1D).

### Performance of serum tau and NfL as diagnostic markers

**Figure 2:**
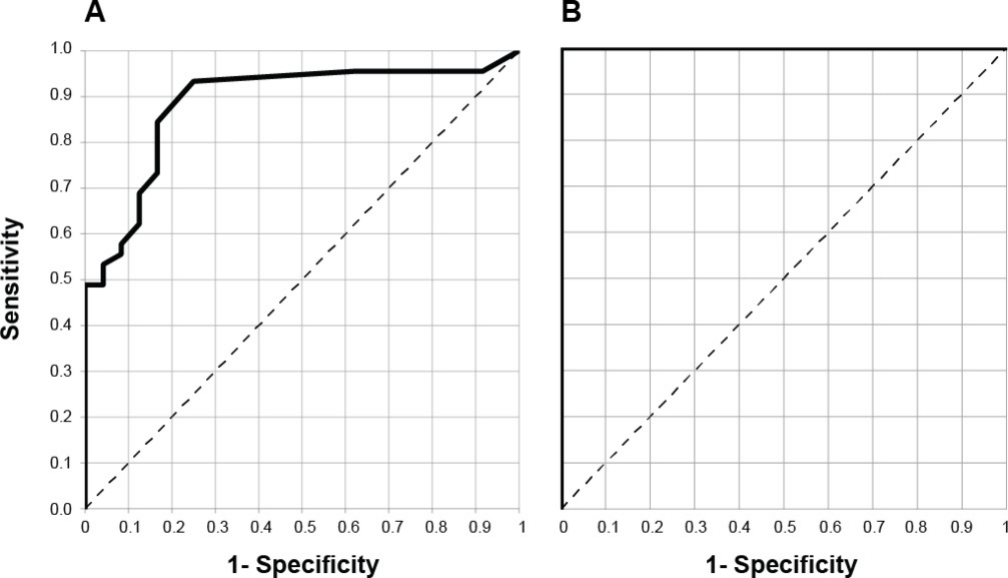
Receiver operating characteristic (ROC) curves for serum tau (A) and neurofilament light (NfL) (B) as tests for distinguishing patients with sporadic Creutzfeldt-Jakob disease (sCJD) from healthy controls.

Receiver operating characteristic curves for serum tau and NfL as diagnostic tests for distinguishing sCJD cases from healthy controls are shown in figure 2. Area under the curve for tau is 0.905 (95% CI 0.826 to 0.984) and for NfL is 1: all patient samples had higher NfL concentrations than all healthy control samples.

A recent study by Steinacker *et al* 30 using Simoa to measure the same biomarkers proposed diagnostic cut-off values of 2.2 pg/mL for serum tau and 44.7 pg/mL for serum NfL for distinguishing CJD cases from controls. We have applied these cut-offs to our results. For tau, this gives a sensitivity of 91% (95% CI 78.8% to 97.5%) and specificity of 83% (95% CI 62.6% to 95.3%). For NfL, this gives a sensitivity of 100% (95% CI 91.6% to 100%) and specificity of 100% (95% CI 85.8% to 100%). This provides some independent validation of the diagnostic cut-off values suggested in Steinacker *et al*,^30^ although it should be noted that our current study included only healthy controls.

### Correlation with rate of disease progression

**Figure 3:**
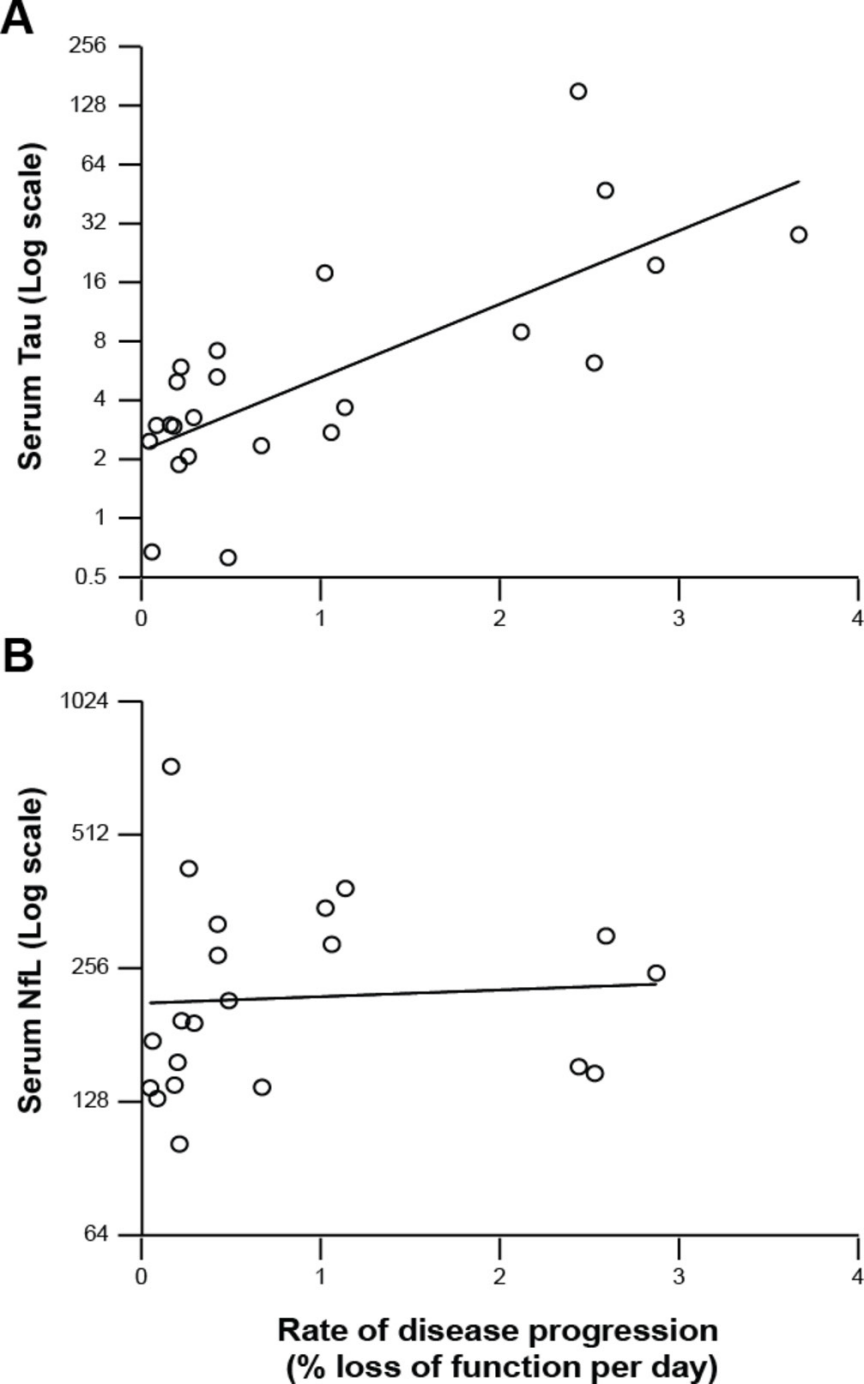
Relationship of serum tau (A) and neurofilament light (NfL) (B) (shown on log scales) to rate of disease progression (quantified as modelled linear slope of functional decline from MRC Prion Disease Rating Scale (MRC Scale) data, as described in Mead *et al*’s^9^ study). Log-tau was positively correlated with rate of disease progression (r^2^=0.552, P<0.001), while log-NfL showed no significant correlation (r^2^=0.0044, P=0.77).

The relationship of serum tau and NfL to rate of clinical disease progression (quantified as modelled linear slope of functional decline from MRC Scale data, as described in Mead *et al*’s^9^ study) is shown in figure 3. Log-tau was positively correlated with rate of disease progression (modelled MRC Scale linear slope): R^2^=0.552, P<0.001. Log-NfL showed no evidence of correlation with rate of disease progression: R^2^=0.004, P=0.77. In the same subset of samples, neither log-tau nor log-NfL correlated with age, gender or number of days between sampling and patient’s death. Log-tau was correlated with *PRNP* codon 129 (as it was in the full sample set—see above), but this was less strong (R^2^=0.3429, P=0.015) than the correlation with rate of disease progression.

### Testing of serial sample sets from individual patients with sCJD

**Figure 4:**
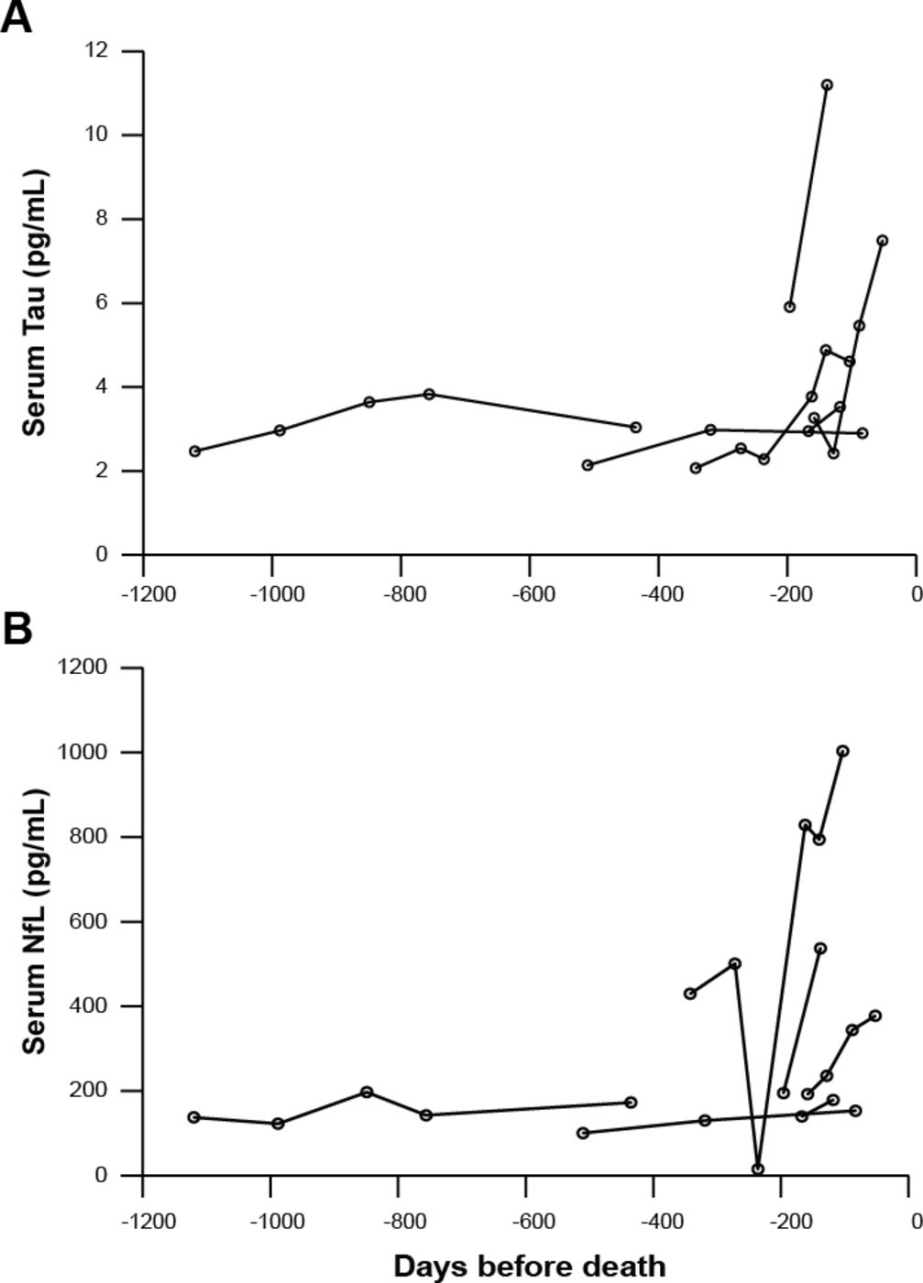
Changes in serum tau (A) and neurofilament light (NfL) (B) concentration over time in six individual patients with sporadic Creutzfeldt-Jakob disease (sCJD). Each line represents an individual patient, and each circle a separate sample. Both tau and NfL concentrations appeared to increase over time in three patients with multiple samples taken within the final 12 months of their illness, and remain stable in two patients with samples taken over longer illness durations.

In six patients with serial sample sets available, both tau and NfL concentrations appeared to increase over time in three patients with multiple samples taken within the final 12 months of their illness, and remain stable in two patients with samples taken over longer illness durations (figure 4). This is presented as a descriptive, exploratory analysis due to the small number of patients and samples. Five of these patients had an MV genotype at *PRNP* codon 129 and one had a VV genotype. It is important to note that this is not a representative subset of the overall sCJD group, being skewed towards those with a slower disease progression and longer clinical durations.^9^

## Discussion

We have shown that concentrations of both tau and NfL are elevated in the serum of patients with sCJD compared with healthy controls. Serum NfL in particular was able to separate patients with sCJD and controls completely, with the lowest sCJD result more than double the highest control. Serum tau, while showing some overlap between patients with sCJD and controls, showed a positive correlation with rate of disease progression, and variation between *PRNP* codon 129 genotypes in keeping with this, suggesting that it might provide a quantitative measure of disease activity. We conclude that both of these proteins represent potentially valuable blood-based biomarkers for sCJD, and warrant further studies to establish whether they may have a useful role in early diagnosis of sCJD, the stratification of disease subtypes, and in monitoring of disease activity or progression, and of response to experimental therapeutic agents.

Our results are closely concordant with those of two other recently published studies which used the same assay platform to test these proteins in serum or plasma from independent populations of patients with sCJD and controls.^30 31^ Both showed elevation of tau and NfL concentrations in patients with sCJD, and one also showed an identical pattern of effects from *PRNP* codon 129 in sCJD to that found in our study.^31^ This common polymorphism is a crucial susceptibility, disease-modifying and strain selection factor in human prion disease.^33^ In our study, NfL was elevated in all patients irrespective of *PRNP* codon 129, while tau was higher in those with the MM genotype. We were also able to investigate correlations with rate of disease progression directly, and show that serum tau, but not NfL, was higher in those with more rapidly progressing disease.

It is tempting to conclude that higher concentrations of serum tau result from rapid neurodegeneration (as seems to be the case for CSF tau,^28^ which has been shown to be strongly correlated with serum tau^31^), raising the possibility that it could provide a quantitative marker of disease ‘intensity’. However, other factors may contribute. For example, patients in the very end stages of their illness at the time of blood sampling were over-represented among those with particularly rapid disease progression and in the codon 129 MM group, and they contributed many of the samples with the highest tau concentrations. It may be that other changes occurring in the preterminal phase, such as breakdown of blood–brain barrier integrity, account for these high concentrations and drive the overall correlation. Adjusting for these potential confounding factors will require a much larger study. PrP^Sc^ type (as defined by Western blotting of protease-resistant prion protein (PrP) from brain homogenate) is known to interact with *PRNP* codon 129 genotype as a phenotype modifier in sCJD.^34^ At the time of publication, PrP^Sc^ type was only available for 40% of patients included in the current study, so it was not appropriate for us to include this in our analysis as the number of cases with each type would be very small. Again, demonstrating any effect of PrP^Sc^ type on serum tau and NfL over and above that of *PRNP* codon 129 (which we have found has a strongly dominant effect on rate of disease progression^9^) would require a larger study.

Our study provides other novel insights. Longitudinal changes in tau and NfL in sCJD have not previously been studied, and the serial sample sets that we tested, although in a small number of patients, show potentially interesting patterns. In patients with multiple samples taken within 12 months of death, there appeared to be a trend towards increasing concentrations of both tau and NfL over this time, raising the possibility of a role as biomarkers of disease progression. In two patients with relatively prolonged disease duration and samples taken over a longer period, the biomarkers appeared to remain quite stably elevated throughout this phase of their illnesses. To establish whether the biomarkers follow different trajectories in different patient subgroups or in different phases of the disease in individual patients will require further data from a larger number of prospectively studied patients.

NfL concentration was very substantially elevated even in patients at an early stage of disease with very mild functional impairment, suggesting that it could have a useful role in early diagnosis. While obtaining blood samples from patients with sCJD prior to the onset of symptoms is rarely possible, carriers of *PRNP* mutations causing inherited forms of prion disease can provide blood samples in the presymptomatic phase. In Steinacker *et al*’s recent study, one serum sample from a presymptomatic carrier of the P102L *PRNP* mutation was found to have a modestly raised NfL concentration.^30^ This will certainly warrant further investigation, as a biomarker of impending disease onset could be extremely valuable in planning therapeutic trials for these patients. Results supporting this role for serum NfL have recently been presented for Huntington’s disease^32^ and familial Alzheimer’s disease.^35^

The relatively small size of this study limits the conclusions that can be drawn beyond the primary aim of comparing sCJD samples and controls. The matching of patient and control groups with respect to factors that might influence biomarker concentrations was not perfect: in particular, age, which is known to affect serum NfL, was lower in the control group than in the patients with sCJD. However, the age ranges in cases and controls overlapped substantially, and our linear regression analyses showed that differences between controls and patients with sCJD remained after adjustment for age.

Clearly, comparing sCJD patient samples with those from healthy controls has limited direct relevance to clinical diagnosis, as in practice patients must be distinguished from those with other diagnoses causing similar clinical presentations. This is particularly relevant for biomarkers such as these which are likely to behave as surrogate markers of neurodegeneration, and indeed are known to be elevated in several other neurodegenerative conditions^18 24 36 37^ (although not to the same levels seen in patients with sCJD in our study), rather than being directly related to specific aspects of the pathology of sCJD.

We have independently replicated the findings of other recent studies,^30 31^ demonstrating the potential of NfL and tau as biomarkers in sCJD, and generated important hypotheses that can be tested in a larger sample archive. The availability of patient blood samples associated with detailed and contemporaneous clinical information collected in the context of a systematic prospective clinical study will provide a remarkable opportunity to fully explore the factors affecting these biomarkers, and define their utility for clinical practice and research.

## References

[1] Collinge J, Gorham M, Hudson F, et al Safety and efficacy of quinacrine in human prion disease (PRION-1 study): a patient-preference trial. Lancet Neurol 2009;8:334–44. 10.1016/S1474-4422(09)70049-3 19278902 PMC2660392

[2] Varges D, Manthey H, Heinemann U, et al Doxycycline in early CJD: a double-blinded randomised phase II and observational study. J Neurol Neurosurg Psychiatry 2017;88:119–25. 10.1136/jnnp-2016-313541 27807198 PMC5284486

[3] Geschwind MD, Kuo AL, Wong KS, et al Quinacrine treatment trial for sporadic Creutzfeldt-Jakob disease. Neurology 2013;81:2015–23. 10.1212/WNL.0b013e3182a9f3b4 24122181 PMC4211922

[4] Haïk S, Marcon G, Mallet A, et al Doxycycline in Creutzfeldt-Jakob disease: a phase 2, randomised, double-blind, placebo-controlled trial. Lancet Neurol 2014;13:150–8. 10.1016/S1474-4422(13)70307-7 24411709

[5] Burchell JT, Panegyres PK Prion diseases: immunotargets and therapy. Immunotargets Ther 2016;5:57–68. 10.2147/ITT.S64795 27529062 PMC4970640

[6] Klyubin I, Nicoll AJ, Khalili-Shirazi A, et al Peripheral administration of a humanized anti-PrP antibody blocks Alzheimer’s disease Aβ synaptotoxicity. J Neurosci 2014;34:6140–5. 10.1523/JNEUROSCI.3526-13.2014 24790184 PMC4004804

[7] Collinge J Mammalian prions and their wider relevance in neurodegenerative diseases. Nature 2016;539:217–26. 10.1038/nature20415 27830781

[8] Thompson AG, Lowe J, Fox Z, et al The Medical Research Council prion disease rating scale: a new outcome measure for prion disease therapeutic trials developed and validated using systematic observational studies. Brain 2013;136:1116–27. 10.1093/brain/awt048 23550114

[9] Mead S, Burnell M, Lowe J, et al Clinical trial simulations based on genetic stratification and the natural history of a functional outcome measure in Creutzfeldt-Jakob disease. JAMA Neurol 2016;73:447–55. 10.1001/jamaneurol.2015.4885 26902324 PMC5701731

[10] Zanusso G, Fiorini M, Ferrari S, et al Cerebrospinal fluid markers in sporadic Creutzfeldt-Jakob disease. Int J Mol Sci 2011;12:6281–92. 10.3390/ijms12096281 22016658 PMC3189782

[11] Zanusso G, Monaco S, Pocchiari M, et al Advanced tests for early and accurate diagnosis of Creutzfeldt-Jakob disease. Nat Rev Neurol 2016;12:427 10.1038/nrneurol.2016.92 27313104

[12] Zerr I, Kallenberg K, Summers DM, et al Updated clinical diagnostic criteria for sporadic Creutzfeldt-Jakob disease. Brain 2009;132:2659–68. 10.1093/brain/awp191 19773352 PMC2759336

[13] Atarashi R, Satoh K, Sano K, et al Ultrasensitive human prion detection in cerebrospinal fluid by real-time quaking-induced conversion. Nat Med 2011;17:175–8. 10.1038/nm.2294 21278748

[14] McGuire LI, Peden AH, Orrú CD, et al Real time quaking-induced conversion analysis of cerebrospinal fluid in sporadic Creutzfeldt-Jakob disease. Ann Neurol 2012;72:278–85. 10.1002/ana.23589 22926858 PMC3458796

[15] Cramm M, Schmitz M, Karch A, et al Stability and Reproducibility Underscore Utility of RT-QuIC for Diagnosis of Creutzfeldt-Jakob Disease. Mol Neurobiol 2016;53:1896–904. 10.1007/s12035-015-9133-2 25823511 PMC4789202

[16] Sandberg MK, Al-Doujaily H, Sharps B, et al Prion propagation and toxicity in vivo occur in two distinct mechanistic phases. Nature 2011;470:540–2. 10.1038/nature09768 21350487

[17] Sandberg MK, Al-Doujaily H, Sharps B, et al Prion neuropathology follows the accumulation of alternate prion protein isoforms after infective titre has peaked. Nat Commun 2014;5:4347 10.1038/ncomms5347 25005024 PMC4104459

[18] Zetterberg H, Wilson D, Andreasson U, et al Plasma tau levels in Alzheimer’s disease. Alzheimers Res Ther 2013;5:9 10.1186/alzrt163 23551972 PMC3707015

[19] Mattsson N, Zetterberg H, Janelidze S, et al Plasma tau in Alzheimer disease. Neurology 2016;87:1827–35. 10.1212/WNL.0000000000003246 27694257 PMC5089525

[20] Tzen KY, Yang SY, Chen TF, et al Plasma Aβ but not tau is related to brain PiB retention in early Alzheimer’s disease. ACS Chem Neurosci 2014;5:830–6. 10.1021/cn500101j 25054847

[21] Randall J, Mörtberg E, Provuncher GK, et al Tau proteins in serum predict neurological outcome after hypoxic brain injury from cardiac arrest: results of a pilot study. Resuscitation 2013;84:351–6. 10.1016/j.resuscitation.2012.07.027 22885094

[22] Olivera A, Lejbman N, Jeromin A, et al Peripheral Total Tau in Military Personnel Who Sustain Traumatic Brain Injuries During Deployment. JAMA Neurol 2015;72:1109–16. 10.1001/jamaneurol.2015.1383 26237304

[23] Meeter LH, Dopper EG, Jiskoot LC, et al Neurofilament light chain: a biomarker for genetic frontotemporal dementia. Ann Clin Transl Neurol 2016;3:623–36. 10.1002/acn3.325 27606344 PMC4999594

[24] Rohrer JD, Woollacott IO, Dick KM, et al Serum neurofilament light chain protein is a measure of disease intensity in frontotemporal dementia. Neurology 2016;87:1329–36. 10.1212/WNL.0000000000003154 27581216 PMC5047041

[25] Piehl F, Kockum I, Khademi M, et al Plasma neurofilament light chain levels in patients with MS switching from injectable therapies to fingolimod. Mult Scler 2017:135245851771513 10.1177/1352458517715132 28627962

[26] Disanto G, Barro C, Benkert P, et al Serum Neurofilament light: A biomarker of neuronal damage in multiple sclerosis. Ann Neurol 2017;81:857–70. 10.1002/ana.24954 28512753 PMC5519945

[27] Arendt T, Stieler JT, Holzer M Tau and tauopathies. Brain Res Bull 2016;126:238–92. 10.1016/j.brainresbull.2016.08.018 27615390

[28] Cohen OS, Chapman J, Korczyn AD, et al CSF tau correlates with CJD disease severity and cognitive decline. Acta Neurol Scand 2015 10.1111/ane.12441 26014384

[29] Noguchi-Shinohara M, Hamaguchi T, Nozaki I, et al Serum tau protein as a marker for the diagnosis of Creutzfeldt-Jakob disease. J Neurol 2011;258:1464–8. 10.1007/s00415-011-5960-x 21360196

[30] Steinacker P, Blennow K, Halbgebauer S, et al Neurofilaments in blood and CSF for diagnosis and prediction of onset in Creutzfeldt-Jakob disease. Sci Rep 2016;6:38737 10.1038/srep38737 27929120 PMC5144074

[31] Kovacs GG, Andreasson U, Liman V, et al Plasma and cerebrospinal fluid tau and neurofilament concentrations in rapidly progressive neurological syndromes: a neuropathology-based cohort. Eur J Neurol 2017;24:1326–e77. 10.1111/ene.13389 28816001

[32] Byrne LM, Rodrigues FB, Blennow K, et al Neurofilament light protein in blood as a potential biomarker of neurodegeneration in Huntington’s disease: a retrospective cohort analysis. Lancet Neurol 2017;16:601–9. 10.1016/S1474-4422(17)30124-2 28601473 PMC5507767

[33] Collinge J, Clarke AR A general model of prion strains and their pathogenicity. Science 2007;318:930–6. 10.1126/science.1138718 17991853

[34] Hill AF, Joiner S, Wadsworth JD, et al Molecular classification of sporadic Creutzfeldt-Jakob disease. Brain 2003;126:1333–46. 10.1093/brain/awg125 12764055

[35] Weston PSJ, Poole T, Ryan NS, et al Serum neurofilament light in familial Alzheimer disease: A marker of early neurodegeneration. Neurology 2017;89:2167–75. 10.1212/WNL.0000000000004667 29070659 PMC5696646

[36] Zhou W, Zhang J, Ye F, et al Plasma neurofilament light chain levels in Alzheimer’s disease. Neurosci Lett 2017;650:60–4. 10.1016/j.neulet.2017.04.027 28428015

[37] Lu CH, Macdonald-Wallis C, Gray E, et al Neurofilament light chain: A prognostic biomarker in amyotrophic lateral sclerosis. Neurology 2015;84:2247–57. 10.1212/WNL.0000000000001642 25934855 PMC4456658

